# Cognitive behavioural group therapy as addition to psychoeducation and pharmacological treatment for adolescents with ADHD symptoms and related impairments: a randomised controlled trial

**DOI:** 10.1186/s12888-022-04019-6

**Published:** 2022-06-02

**Authors:** Anne-Lise Juul Haugan, Anne Mari Sund, Susan Young, Per Hove Thomsen, Stian Lydersen, Torunn Stene Nøvik

**Affiliations:** 1 Department of Mental Health, Faculty of Medicine and Health Sciences, Regional Centre for Child and Youth Mental Health and Child Welfare (RKBU), NTNU- Norwegian University of Science and Technology, Trondheim, Norway; 2 Department of Child and Adolescent Psychiatry, St. Olav University Hospital, Trondheim, Norway; 3 Psychology Services Limited, London, UK; 4 Department of Psychology, University of Reykjavik, Reykjavik, Iceland; 5 Department of Child and Adolescent Psychiatry, Aarhus University Hospital, Aarhus, Denmark

**Keywords:** Attention-deficit/hyperactivity disorder,, Adolescence,, Cognitive behavioural therapy,, Group therapy,, Randomized controlled trial

## Abstract

**Background:**

Cognitive behavioural therapy (CBT) is recommended for attention-deficit/hyperactivity-disorder (ADHD) in adolescents. However, all CBTs are not created equal, and the guidelines do not specify which CBT interventions are the most effective for this patient group. This study examines the efficacy of a group CBT without parent involvement as follow-up treatment compared to no additional CBT in adolescents with persistent and impairing ADHD symptoms after a short psychoeducational intervention and medical treatment.

**Methods:**

The authors conducted a two-arm parallel randomized controlled trial in two child and adolescent mental health outpatient clinics in Norway. One hundred patients aged 14–18 years with a diagnosis of ADHD (66%) or subthreshold ADHD (34%) were randomized to either a 12-week group CBT program (*N* = 50) or a non-CBT control condition (*N* = 50). Assessments were made at admission to the clinic, two weeks before and two weeks after treatment. The primary outcomes were parent-, teacher- and self-ratings of ADHD symptoms (ADHD Rating Scale-IV), and the secondary outcomes were ratings of ADHD symptom severity, executive function, functional impairment, and emotional problems. Evaluators blinded to group allocation rated ADHD symptom severity with the Clinical Global Impression Scale for Severity (CGI-S) at baseline and post-treatment.

**Results:**

Analyses using mixed-effects models showed no difference between the treatment arms from baseline to post treatment in primary and secondary outcomes.

**Conclusions:**

Contrary to our hypothesis, we found no incremental treatment effect on the part of a group CBT as follow-up to psychoeducation and pharmacological treatment on ADHD symptoms and accompanying impairments. Limitations with the CBT was the large number and low dosage of treatment components, causing restricted time for practice. Unlike evidence-based, individualized targeted CBTs with parent involvement, a group CBT directed solely at the adolescents with no parent involvement does not appear effective for treating ADHD.

**Trial registration:**

NCT02937142, 18/10/2016.

**Supplementary Information:**

The online version contains supplementary material available at 10.1186/s12888-022-04019-6.

## Background

Attention-deficit/hyperactivity disorder (ADHD) is a neurodevelopmental disorder characterised by levels of inattention, hyperactivity and impulsivity that lead to impairment [1]. In adolescence, ADHD is often associated with a range of social and emotional sequelae, including anxiety, depression, interpersonal difficulties, low self-esteem, low academic achievement, and substance abuse [2–5]. Although medication may be effective in reducing ADHD’s core symptoms [6], this treatment alone may not be sufficient to remediate ADHD and its associated conditions. Some patients experience adverse side effects or do not respond well to medical treatment [7], the long-term effect of pharmacotherapy is inconclusive [8] and many adolescents discontinue treatment in the transition to adulthood [9, 10]. Because ADHD often persists across the lifespan [11], there is a need for additional treatments to learn strategies and skills for coping with impaired executive functioning and functional impairments. This seems especially imperative for adolescents, who are at a crossroads, with expectations of parental detachment and increased independence on the one hand and a need for external structure and emotional support on the other hand. The National Institute for Health and Care Excellence (NICE) guidelines recommend multimodal treatment for children and young adults with ADHD [12]. This includes ADHD-focused support, including education and information about the causes and effects of ADHD, advice on parenting strategies and supportive measures in school. Pharmacotherapy is recommended if ADHD symptoms persist after environmental modifications. In addition, cognitive behavioural therapy (CBT) is recommended as a treatment option for young people if symptoms remain impairing after pharmacological treatment [12]. A limitation with this recommendation however, is that all CBTs are not created equal, and the guidelines do not specify which CBT programs to use for young people with ADHD [12, 13]. Systematic reviews of psychosocial interventions directed at children and adolescents show that most interventions combine components from behaviour therapy/behaviour contingency management, cognitive restructuring techniques and skills training, to reduce symptoms of ADHD and its` associated impairments [6, 14]. Compared to the childhood treatments that involve parents to a large extent, treatments directed at adolescents have a more moderate parent involvement, and they include more individualized engagement components, as well as skills training compared to the childhood treatments. According to Evans et al. [14, 15], behaviour management treatments including behavioural parent training, behaviour classroom management and behavioural peer interventions are considered well-established treatments for children with ADHD. For adolescents, only organization training has been considered well established. CBT programs directed at adolescents and their parents have so far been considered as probably efficacious [14], but preliminary results have been promising [16, 17].

The CBTs targeting adolescents with ADHD may be divided into school-based and clinic-based treatments. Evans and colleagues developed the Challenging Horizons Program (CHP), a school-based training intervention to help young adolescents with ADHD improve their inattention, social and scholastic skills [18]. In the CHP, the adolescents meet twice a week for about 2h after school, across one academic year. The program also includes three parent meetings. In a randomised trial, the participants demonstrated significant improvements in parent-rated organization and time management skills, homework completion, and ADHD inattention symptoms, but not social skills, compared to participants in two control conditions [19]. Another school-based intervention developed by Langberg and colleagues is the Homework, Organization, and Planning Skills (HOPS) program [20]. This intervention which is delivered during the school day by school mental health providers, aims to improve organizational skills and homework problems in middle school students with ADHD. It includes 16 short sessions (20 minutes) over an 11-week period. Parents are included in two of the sessions. A randomized study comparing participants receiving HOPS to a waitlist control group demonstrated significant improvements on parent-, but not on teacher- ratings of materials management, planning skills, and homework completion in favour of HOPS [21].

Sibley and colleagues developed a clinic-based skills intervention for adolescents with ADHD between the ages of 11 to 15 [22]. The Supporting Teens` Autonomy Daily (STAND) program is a modular treatment with 10 1-hour parent-teen sessions with a menu of skills that can be targeted (e.g, organization, time management, test taking and note taking) from which the family selects four to address. Parent- teen contracts are used, in which parents provide behavioural contingencies based on the adolescents’ use of the targeted skills at home and school to facilitate the skills. Motivational interviewing (MI) is integrated to enhance treatment engagement. Results from both a pilot study and a randomized trial revealed significant improvements in parent-, but not teacher- rated, ADHD symptom severity, planning and organizational skills, as well as parenting stress compared to a treatment as usual control group [17, 22]. Another promising CBT program for adolescents with ADHD was conducted by Sprich and colleagues [16]. This CBT, originally developed for adults with ADHD [23] was conducted with medicated adolescents between 14 to 18 years. The 12-session program which also involves the parents in two of the sessions, includes three modules focusing on psychoeducation, cognitive restructuring techniques and training in organisation and planning skills. A randomized trial revealed significantly reduced parent- and adolescent-rated symptom severity and reduced ADHD symptoms in the control group compared to a waitlist control group, demonstrating initial efficacy of CBT for adolescents [16]. Furthermore, two short-term CBT interventions targeting adolescents with ADHD was developed by Boyer and colleagues. Both interventions include elements from MI in combination with either planning skills (Plan My Life) or a Solution-Focused Treatment. The programs consist of eight adolescent-sessions and two parental-sessions [24]. A comparison of the programs in an ADHD population aged 12 to 17 years revealed reduced parent-rated ADHD symptoms, planning problems and improved executive functions in both treatment arms. A limitation of this study was the lack of waitlists or treatment as usual control group.

The first RCT to examine the efficacy of group CBT on a sample of late adolescents and young adults medicated for ADHD was conducted by Vidal et al. [25]. Different from the previous clinic-based CBTs that involve parents to various extent, this was a patient focused 12-session multicomponent CBT program based on psychoeducation and cognitive behavioural principles to facilitate skills related to impulsivity, emotion regulation, interpersonal skills, planning strategies and techniques to improve inattention using MI techniques. The study showed beneficial effects on both parent- and self-rated ADHD symptoms and parent rated functional impairment as compared to a waitlist control condition. One limitation of the study was the exclusion of patients with comorbid emotional disorders, which are common in this patient group [4, 26, 27]. Similar to the CBT programs of both Vidal and Sprich, the Young-Bramham program (YBP) incorporates elements from psychoeducation, structured skills training and cognitive behavioural therapy to target ADHD core symptoms as well as comorbid problems. The program is modular based, and the choice of modules and number of sessions may be adjusted to fit the needs of the individual patient or group participants [28]. In addition to cognitive restructuring techniques, the YBP includes strategies to improve attention and memory functions, it includes skills training in planning and organization and incorporates behavioural techniques such as graded task assignments, modelling and roleplay to improve social regulation and communication [28]. The YBP program has not previously been studied in an adolescent population, but Bramham and colleagues studied the effect of a short and intensive YBP group program with ADHD adults, which revealed promising preliminary results with significantly greater improvement on measures of knowledge about ADHD, self-efficacy, and self-esteem in the CBT group compared to the waitlist control group [29].

When planning a treatment study for adolescents with ADHD, we found no manual suited for the purpose in a Scandinavian language. Inspired by the positive results from the group treatment by Vidal et al. [25], we decided to develop a Norwegian research manual based on selected modules from the YBP in collaboration with one of its authors, SY. After selecting modules from the YBP thought appropriate for our adolescent population, we translated it to Norwegian and tested the manual, the feasibility and acceptability of the program in a pilot study. We refer to Novik and colleagues for the study protocol [30]. We preferred group treatment to individual treatment as the group format provides the opportunity to meet other patients with similar problems which offers normalisation, mutual understanding, and also opportunities to share strategies for coping with problems and acquire news skills in a non-judgemental environment which we consider important for adolescents with ADHD.

The NICE guideline recommends CBT as an additional treatment in ADHD patients who still present impairing symptoms after psychoeducation and pharmacological treatment [12]. To our knowledge, no published studies have examined the efficacy of CBT as follow-up treatment in a sample of ADHD adolescents with and without comorbid emotional disorders who previously received this recommended treatment. The aim of our study was thus to assess the efficacy of an age-adapted group CBT program as additional treatment to a short psychoeducational intervention and medical treatment in adolescents still presenting impairing ADHD symptoms. Based on previous CBT studies on medicated adolescent populations [16, 25] we predicted that the CBT group would be superior in terms of showing incremental improvement in ADHD symptoms, executive functions, and functional impairment compared to a control group having received the same previous interventions as the CBT group, but no additional CBT.

## Methods

### Study design and procedure

**Fig. 1:**
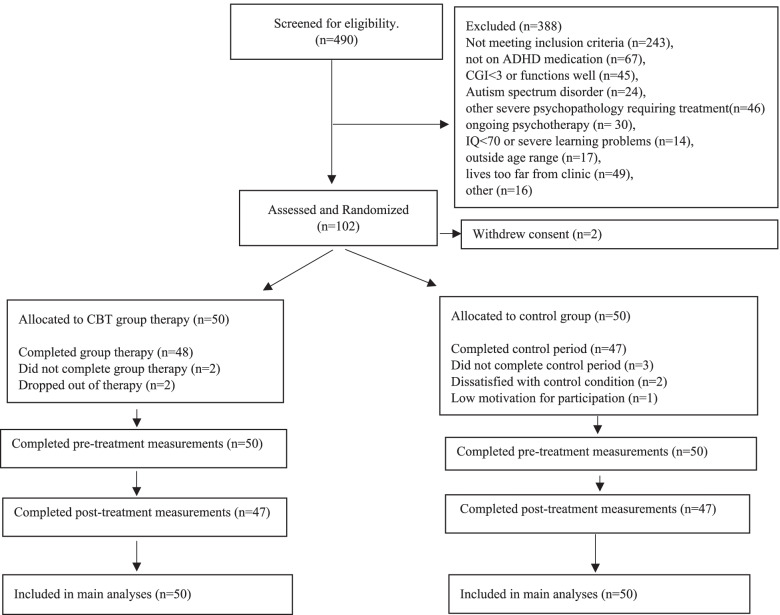
Flow diagram of participants in cognitive behavioural group therapy for adolescents with ADHD – a randomised controlled trial

This was a 12-week, RCT efficacy trial with two study arms comparing CBT group therapy as a follow-up treatment with a passive no additional CBT control condition in a clinical context. A detailed research protocol has been published elsewhere [30]. The recruitment, intervention and data collection were conducted in two outpatient child and adolescent psychiatry (CAP) university clinics in Mid-Norway. Recruitment began in February 2017, and the last follow up data were collected in January 2020. The measures included self-, parent, and teacher reports and were collected while the participants were on medication, two weeks before and two weeks after the intervention. Clinical evaluations of ADHD symptom severity using the Children’s Global Assessment Scale (CGAS) [31] and Clinical Global Impression Scale for Severity (CGI-S) [32] were collected two to four weeks before the intervention and two weeks after the intervention, by clinicians (a clinical psychologist or a child and adolescents psychiatrist) blinded to the participants’ group allocation. Participants were screened for eligibility and recruited from the two CAP outpatient units by the last author in cooperation with the clinicians responsible for this patient group. Participants (*N* = 9) who previously received pharmacotherapy but were unable to continue treatment because of intolerable side-effects or little effect were included in the study for ethical reasons and to achieve enough participants for the study. During recruitment, we included patients with mild to moderate behavioural problems to achieve enough participants. The inclusion of patients with behaviour problems was a deviation from the trial registration but was described in the study protocol [30]. Six participants were recruited through primary care physicians after postings in a local newspaper and advertising via social media. The last author screened these participants before inclusion, and they previously underwent the same diagnostic procedures as well as received psychoeducation and pharmacological treatment in a CAP clinic similar to the other participants before being discharged. Furthermore, they followed the same inclusion criteria as the other participants. All participants and their parents were provided oral and written information about the content of the study and its treatment arms by CAP clinicians. A flowchart for the timeline for the recruitment, follow-up assessments and post-treatment analyses is presented in Fig. 1.

### Participants

The sample characteristics are presented in Table 1. The participants were recruited from a group of adolescents between the ages of 14 to 18, the mean age was 15.3 (SD = 1.3), with a previous clinical diagnosis of ADHD according to the International Statistical Classification of Disease and Related Health Problems (ICD-10) [33]. A clinical psychologist or a child and adolescent psychiatrist made psychiatric diagnoses at the first intake to the CAP clinic (0–13 years). The CAP clinic’s standardised procedure for the assessment and diagnosis of hyperkinetic disorder is based on the national guidelines for the assessment and treatment of ADHD [13], which are similar to the NICE ADHD guidelines [12]. This procedure requires a thorough developmental history, an examination of comorbid psychiatric disorders, a somatic assessment and the use of questionnaires filled out by the adolescents, parents, and teacher informants to obtain ADHD symptom scores (ADHD rating scale). The diagnostic criteria for hyperkinetic disorder in ICD-10 are nearly identical to those of the Diagnostic and Statistical Manual of Mental Disorder 5th edition’s (DSM-5) [1] diagnosis of ADHD combined presentation. The Norwegian ADHD guidelines [13] allow for hyperkinetic disorder to be diagnosed in patients with severe inattentive symptoms only, corresponding to the DSM-5 Inattentive type. Patients receiving an ADHD diagnosis at the CAP clinic are usually offered interventions as described under the subheading “CAP standard clinical intervention” before being transferred to community care. When patients are being referred to the clinic for a follow-up medical treatment and/or associated conditions because of increased symptoms or impairments, and the patient received an ADHD diagnosis at an early time-point, the parents need to confirm ADHD symptoms and clinical impairment in a clinical interview together with the patient at readmission. All the participants received an initial ADHD diagnosis or ADHD symptoms were confirmed by a parent informant within 0 to 5 years before inclusion (Mean = 1.5 years, SD = 1.2). For 94% of the population, the ADHD symptoms were confirmed by a parent rater within the last three years of inclusion. In addition, we interviewed each participant with the Schedule for Affective Disorders and Schizophrenia for school age children-Present and Lifetime Version (Kiddie-SADS-PL) [34] at the CAP units before intake to the study to assess for the presence of ADHD symptoms and psychiatric comorbidities. In cases of diagnostic uncertainty, current comorbidities were checked with the adolescents’ medical record. Ultimately, 66% of the adolescents reported symptoms above threshold for a DSM-5 ADHD diagnosis. Adolescents who reported symptoms below the threshold for ADHD according to the DSM-5 but had impairing ADHD symptoms while on medication (34%) were allowed into the study [30] and were designated as subthreshold ADHD. The participant population’s mean ADHD RS-IV parent total score at the first intake to the CAP clinic was 33.7 (SD = 8.8, *n* = 75), while the mean baseline score before the trial was 25.0 (SD = 8.8, *n* = 97). Ninety-one percent of the participants were on pharmacological treatment for ADHD. Fifty-three percent of the participants had at least one current comorbid condition according to the DSM-5 (see Table 1). Additionally, IQ scores were obtained by using the Wechsler Intelligence Scales for Children (WISC-IV) [35] or Adults (WAIS-IV) [36].

**Table 1:** Clinical characteristics of the participants at baseline (study inclusion)

Characteristics	CBT (*n* = 50)	Control (*n* = 50)
Mean age, years (SD)	15.9 (1.3)	15.8 (1.3)
Male patients (n [%])	21 (42.0)	22 (44.0)
Full-scale IQ (n [mean, SD])	44 (94.3[12.8])	42 (93.4[13.2])
**Parent socioeconomic status (n [%])**	38 (76)	37 (74)
Less than compulsory school or 1–2 years of high school (0–11 years)	13 (34.2)	6 (16.2)
Completed high school and 1 year of training after high school (12–13 years)	3 (7.9)	4 (10.8)
Academy university for up to four years (14–15 years)	15 (39.5)	15 (40.5)
Academy/ University for four years or more (16 years and more)	7 (18.4)	12 (32.4)
**Previous CAP psychosocial treatments (n [%])**		
Webster Stratton, Incredible years	10 (20)	6 (12)
Cognitive behavioral Therapy (CBT)	2 (4)	1 (2)
Routine Clinical Care^a^	18 (36)	24 (48)
Other^b^	4 (8)	4 (8)
**CAP standard clinical intervention (n [%])**		
Short psychoeducational intervention with patient and parents	33 (66)	32 (64)
Short psychoeducational intervention with patient alone	24 (48)	29 (58)
School collaborative meeting	47 (94)	48 (96)
ADHD full day lecture	35 (70)	36 (72)
**ADHD presentation (Kiddie-SADS-PL) (n [%])**		
Predominantly Combined	18 (36.0)	13 (26.0)
Predominantly Inattentive	17 (34.0)	18 (36.0)
Subtreshold ADHD	15 (30.0)	19 (38.0)
**Medication**^**c**^ **(n [%])**		
ADHD medication	44 (88.0)	47 (94.0)
Sleep medication	6 (12.0)	2 (4.0)
Other psychopharmacological treatment	5 (10.0)	2 (4.0)
**Psychiatric comorbidities**^**d**^ **(Kidde-SADS-PL) (n [%])**		
Anxiety disorders	19 (38.0)	18 (36.0)
Posttraumatic stress disorder	0 (0.0)	1 (2.0)
Depressive disorder NOS/Dysthymic disorder	8 (16.0)	3 (6.0)
Obsessive Compulsive Disorder	1 (2.0)	2 (4.0)
Tics disorder or Tourette’s Disorder	4 (8.0)	5 (10.0)
ODD/Disruptive behaviour disorder NOS	6 (12.0)	5 (10.0)
Autism spectrum disorder (mild symptoms)	2 (4.0)	2 (4.0)
**Learning Disorders, reading disorders or mixed (n [%])**	8 (16.0)	10 (20.0)

Note: *Full-scale IQ* Wechsler Intelligence Scale for Children or Adults (WISC-IV, WAIS-IV), *SD* Standard deviation, *ADHD* Attention-deficit/hyperactivity disorder, *ODD* Oppositional Defiant Disorder

^*a*^*Routine clinical care* Supportive therapy for patients and/or parents for mild emotional and behavioural problems

^*b*^*Other* Dialectic behaviour therapy (DBT), eye movement desensitizing and reprocessing (EMDR), habit reversal training (HRT) and family therapy

^*c*^*Medication* ADHD medication includes methylphenidate, lisdexamfetamine, atomoxetine, and guanfacine; *sleep medication*: melatonin; *other pharmacological treatment* includes neuroleptic medication; risperidone, quetiapine; anti-epileptic medication: valproate, lamotrigine

^*d*^*Psychiatric comorbidities* are based on Kiddie-SADS-PL interview with the adolescents and converted to DSM-5 diagnoses

The inclusion criteria were thus a previous full diagnosis of ICD-10 ADHD, a DSM-5 diagnosis of ADHD or subthreshold ADHD, confirmed by the Kiddie-SADS-PL interview, and evidence of clinically impairing symptoms (a Clinical Global Impression Scale for Severity (CGI-S) clinician score of 3 (mildly ill) or greater at baseline). Participants with comorbid diagnosis including mild to moderate depressive disorders, anxiety disorders, bipolar disorders, tic disorders, oppositional defiant disorder and mild degree of autism spectrum disorders were included in the study. All participants needed to have been on a stable pharmacological treatment for ADHD for at least two months prior to randomisation into the study. However, participants who had previously been medicated but terminated treatment because of minimal treatment effect or having experienced intolerable side effects after at least two medication trials were included. The participants could not be seeking or engaged in parallel psychosocial interventions during the study period. A crisis involving the considerable worsening of psychiatric problems (family crisis, worsening of depressive symptoms or aggression/acting out in the home environment) could, however, necessitate a limited supplemental examination or supportive intervention with the parents or the patient. One participant received two extra hours with parental support after acting out at home, and four participants received four supplemental therapy sessions related to depressive symptoms/emotional dysregulation. All of them were part of the control group.

The exclusion criteria were severe depression, suicidal behaviour, conduct disorder, psychoses, intellectual disability (IQ < 70) and current substance abuse. Patients in on-going psychotherapy or previously having received CBT for ADHD (CBT with treatment modules directed at core ADHD symptoms or executive functions as shown in Table 2), and patients not interested in psychopharmacological treatment, were also excluded.

**Table 2:** Contents of the group cognitive-behavioural therapy (CBT) program

	Session	Themes
**Core symptom modules**	1	Orienting participants to the program, including content, structure, and the basic CBT principles. Participants receive psychoeducation about ADHD and write down individual treatment goals.
	2	Attention: Various forms of attention and the impact of motivation, anxiety and stress are introduced and discussed. Various attention control strategies are presented and rehearsed in session.
	3	Memory: The various memory systems are introduced. External and internal memory strategies are presented. Memory games and exercises are practised within group meetings.
	4	Organising and time-management: Consequences of dysfunctional planning and time-management are discussed. Six steps for making a time plan, including use of daily planners and rewards are introduced and rehearsed.
	5	Impulsivity: Consequences of having low self-control are introduced and discussed. Various impulse control strategies, including self-talk and distraction techniques, are presented and rehearsed in the session.
**Comorbid and associated problem modules**	6	Problem solving: The participants learn how to define problems, generate solutions and evaluate them. We rehearse in session, and finally, we evaluate the level of success.
	7	Anxiety: Psychoeducation on basic CBT principles, how to cope with negative thoughts, the three- legged table, relaxation strategies and the role of exposure in changing behaviour.
	8	Depression and sleep management: Introducing the cognitive model of depression, challenging negative thoughts and the positive role of activity. Psychoeducation about sleep and sleep strategies are introduced.
	9	Interpersonal relationships and communication: Introducing and rehearsing verbal and nonverbal communication strategies.
	10	Frustration and anger management: Consequences of bad anger management are discussed. We introduce various management strategies, including self-talk, distraction techniques, reframing the situation and relaxation.
	11–12	Preparing for the future: We present and discuss the challenges of having ADHD in the transition to young adulthood. We repeat some of the highlights from the program and discuss the participants’ future goals and which skills can be used to achieve them.

Note: All sessions include group activities, homework assignments and telephone coaching between sessions. The content is based on the CBT program of Young and Bramham, 2012

In all, 102 patients were randomised, and 100 participants completed the baseline assessments. The two participants who withdrew consent were not included in the analyses. Those who completed measures at baseline but not post-treatment were included in the analyses according to intention-to-treat principles.

### CAP standard clinical intervention

The CAP clinical interventions are conducted shortly after receiving an initial ADHD diagnosis. Sixty-five percent of the participants received a short psychoeducational intervention (1–2 hours) together with his or her parents after receiving an ADHD diagnosis at the CAP clinic, as recommended in the ADHD guidelines [12, 13]. This psychoeducation typically consisted of information about ADHD diagnoses, symptoms, causes and treatment options. It was delivered by the patient’s clinician (a psychologist, medical doctor/child and adolescent psychiatrist or clinical education specialist). Fifty-three percent of the participants received 1–2 individual psychoeducational sessions with their clinician either in addition to the meeting with parents, or as the only psychoeducational intervention received at the CAP clinic. The content of these sessions was not standardized, so the information varied across clinicians and participants. Ninety-two percent of the participants received at least one of these psychoeducational interventions. The patient’s parents and schoolteachers had a collaborative meeting with the CAP clinician and/or a clinical education specialist to inform about the ADHD diagnosis and discuss individualised supportive measures in school (1 hour). Parents and a schoolteacher were also offered a standardized full-day lecture, with information about ADHD, pharmacotherapy, psychosocial interventions (help with planning and organising, supportive communication and the use of helping aids), and school interventions (regular daily routines, the use of a daily plan and week plans in school, clear communication/ short messages, and the use of rewards). These lectures are delivered by various ADHD specialists. All the families of the participants received at least one of these psychoeducational interventions. See Additional file 1 for more comprehensive information about the content.

Patients with persistent ADHD symptoms after receiving psychoeducation and a limited supportive school intervention were offered pharmacological treatment according to National clinical guidelines for ADHD [13]. Documents included in the hospital quality system (EQS) give detailed procedures for beginning and evaluating treatment. Methylphenidate is first drug of choice, while amphetamine or atomoxetine are second choices. The ADHD rating scale [37] was used as systematic effect measure during the titration trial using both parent and teacher ratings. In addition, the patient or his or her parents completed an adverse reaction form. Clinicians mapped specific problematic ADHD symptoms before beginning medication and considered improvement in symptoms and function in everyday life and any significant side effects during the evaluation. A second or third trial was indicated if the first drug was ineffective or caused significant side-effects. See Additional file 2 for information on participant medication type and dosage.

### CBT intervention

The first and last author developed the CBT treatment manual in collaboration with Dr. Susan Young. It is based on the “Young Bramham programme” which is a CBT program developed for adolescents and adults with ADHD and comorbid symptoms by Susan Young and Jessica Bramham [28]. The YBP includes information on ADHD, the principles of CBT and strategies for managing core ADHD symptoms, such as inattention and memory problems, impulsivity, and organization and time management issues. Modules with strategies for problem-solving, interpersonal problems, anxiety, depression, frustration, and anger management were also included, as these are common problem areas in our patient group. Our CBT program was adapted to fit a 12-week group format with 90-minute sessions (see Table 2 for the main contents of the program and Additional file 3 for a more detailed description of the program). Basic CBT elements including the ABC model with the triangulation of thoughts, feelings, and behaviour, identifying dysfunctional thoughts/cognitive restructuring techniques, Socratic questioning and positive reinforcement were used throughout the program. All the sessions were structured using the same format, with psychoeducation, group discussions, skills training, role-play, and individualised weekly home assignments. The language, in terms of the material and choice of modules, was adapted to fit an adolescent ADHD population with comorbid disorders. A PowerPoint presentation was developed for a visual presentation of the material, and the participants received accompanying handouts containing the main content of the modules. The groups consisted of 4–6 participants and were conducted by two clinicians recruited from the clinic (a clinical psychologist, a child and adolescent psychiatrist/and or a clinical education specialist). All the group leaders had experience with CBT, but only one was a CBT therapist. All the group leaders were trained before delivering the intervention. The training included a full day course on CBT and delivering of the research treatment manual before the intervention. They were also given a copy of the Young-Bramham textbook describing treatment strategies in CBT for ADHD. We refer to Andersen et al. for supplemental background information on the group leaders [38].

The group leaders registered the attendance of each participant. Parents were not involved in the program. A research assistant telephoned the participants every week, reminding them of their home assignment; evaluated medical adherence and verified that they did not receive any other type of psychological treatment. One routine medical follow-up was usually performed during the intervention period. This consultation involved a child and adolescent psychiatrist evaluating general health status, the side effects of medication and blood pressure, heart rate and weight. The patient was encouraged to report any difficulties related to the medication since the last consultation.

### Control group

The participants in the control group continued medical treatment and received one routine medical follow-up (as in the CBT group). This was a passive control condition with no additional intervention received after the CAP standard intervention. A research assistant contacted them once a week to monitor medication adherence and verify that no other psychological treatment was received. The participants were not offered entry into a CBT group after the post-intervention assessments. They could, however, engage in other treatments according to their clinical needs after completing the post-treatment assessments.

### Fidelity

Continuous CBT supervision was given to the group leaders on a nearly weekly basis by an experienced CBT supervisor (AMS), whereby the therapists could receive guidance and support for upcoming sessions to stay adherent to the method. AMS also attended some sessions as an observer.

All sessions were videotaped, and adherence to the manual and CBT core principles relevant to the study was rated based on a random selection of 20 sessions (22%) and stratified by early [2–7] and late [7–11] sessions by an external clinician experienced with group CBT. The Competence and Adherence Scale for Cognitive Behavioural Therapy (CAS-CBT) [39] covers basic CBT components, as well as specific session goals that can be adapted to fit a specific treatment. A minimum score of 3 is considered adequate for both manual adherence and therapist competence. Treatment fidelity was acceptable across all measures, including adherence related to the CBT content (M = 3.38, SD = 0.75), program adherence (M = 3.47, SD = 0.69) and the CBT competence score (M = 3.25, SD = 0.87).

Medication adherence was assessed by telephone, specifically interviewing the participants on a weekly basis during the intervention period. The participants were asked about what medication they were on, the dosage and whether they had used the medication as prescribed during the last week.

Inter-rater reliability was calculated for the CGAS scores using the intraclass correlation coefficient (ICC) at baseline. The last author (TSN) scored a random sample of 20 participants (20%) originally scored by the first author (AJH) based on the written records of the participant interviews. The ICC was 0.78 (95% CI 0.53 to 0.91). Three other child and adolescent psychiatrists experienced in the assessment of ADHD scored the CGAS post-treatment. A random sample of 18 (19%) of the participants was simultaneously scored by TSN at this time. The ICC for the CGAS ratings was 0.92 (95% CI 0.80 to 0.97).

The CGI-S scores were based on short interviews with the adolescent and a parent and set by TSN at baseline and three other child and adolescent psychiatrists post treatment. The child and adolescent psychiatrists scoring CGI-S with the adolescent and parent post-treatment scored the CGAS at the same time. A random sample of 16 (17%) of the participants was scored simultaneously by TSN. Cohen’s weighted quadratic kappa for the CGI-S ratings was 0.78 (95% CI 0.54 to 1.00).

### Measures

See Table 3 for an overview of the various outcome measures with different informants at different time points. All the questionnaires (except for the teacher reports) were filled out at the CAP clinic under the surveillance of a research assistant. Psychiatric diagnoses were assessed using the Schedule for Affective Disorders and Schizophrenia for school-age children-Present and Lifetime Version (Kiddie-SADS-PL) [34]. The instrument covers DSM-IV psychiatric diagnosis for school-age children (age 7–17), and the findings suggest that it generates reliable and valid child psychiatric diagnoses [34].

**Table 3:** Instruments used with various informants during time points in the trial

Instruments used in the trial	Baseline	Post-treatment
Kiddie-SADS-PL psychiatric interview (A)	x	
Primary measures		
ADHD RS-IV (ADHD symptoms) (P, S, T)	x	x
Secondary measures on functional impairment		
Children’s Global Assessment Scale (CGAS) (C) (A, A + P)^*^	x	x
Clinical Global Impression (CGI) (C) (A + P)	x	x
Weiss Functional Impairment Rating Scale (WFIRS) (P, S, T)	x	x
Secondary measures of executive functions		
BRIEF (Executive functions) (P, S, T)	x	x
Secondary measures of emotional functions		
SCARED (Anxiety) (S)	x	x
Short Mood and Feelings Questionnaire (MFQ) (S)	x	x
General Perceived Self-Efficacy Scale (S)	x	x
Rosenberg Self-Esteem scale (S)	x	x
Adolescents Sleep Wake Scale (ASWS) (S)	x	x

*Note Baseline* study inclusion, *Post-treatment* 12-week assessment, *A* Adolescent participant, *C* Clinical evaluation, *P* Parent-report, *S* Self-report, *T* Teacher-report, *ADHD* Attention deficit hyperactivity disorder, *BRIEF* Behaviour Rating Inventory of Executive Function. *Only participant A at baseline and A and P post-treatment

### Primary outcomes

ADHD symptoms were assessed using parent, teacher, and self-ratings on the *ADHD Rating Scale* (ADHD RS-IV) [37, 40]. The questionnaire contains an 18-item scale corresponding to the diagnostic criteria for ADHD and rates the frequency of each item from 0 = not at all to 3 = very often, with higher scores indicating more symptoms. The scale consists of nine symptoms of inattention and nine symptoms of hyperactivity, which represents two subscales, in addition to a total score. The scale has been validated for children and adolescents (age 5–18) with ADHD, with adequate reliability and validity [40]. A pan-European study found strong evidence for cross-cultural factorial validity, internal consistency as well as convergent and divergent validity supporting use of the ADHD-RS-IV across European countries [41]. In the current study, the Cronbach alpha coefficients were 0.78 to 0.81 on the ADHD-RS IV parent report, 0.80 to 0.82 for teacher ratings, and 0.80 to 0.84 for self-ratings.

### Secondary outcomes

The *Clinical Global Impression Scale for Severity* (CGI-S) [32] was used to rate the severity of a patient’s illness related to ADHD symptoms. This rating is based on observed and reported symptoms, behaviour, and function in the past seven days. It is a 7-point scale ranging from 1 = normal, meaning not at all ill, 3 = mildly ill, to 7 = among the most extremely ill patients, with 0 = not assessed. Higher scores indicate more severe ADHD symptoms. This scale is often used in psychopharmacological research and has shown to have adequate sensitivity in drug trials [32].

The *Children’s Global Assessment Scale (CGAS)* [31] is a numeric scale used to measure the general psychosocial functioning of children under the age of 18 during the last month. The range is from 1 (lowest function) to 100 (excellent function). The Norwegian version has shown acceptable convergent, discriminant and predictive validity as well as acceptable interrater reliability [42].

*The Weiss Functional Impairment Rating Scale parent and self-report* (WFIRS-P, WFIRS-S) [43] consist of 50 and 69 items, respectively, divided into six and seven domains of impairment that are typically affected in ADHD (family, school and learning, life skills, self-concept, social activities and risky activities). Items range from 0 = not at all to 3 = very often, with 4 = not applicable, with higher scores indicating more impairment. We used the mean total score in this study, which represented the mean of all the subscales. The Norwegian version has shown acceptable psychometric properties in an adolescent ADHD population [44]. In this study, the Cronbach alpha coefficients for the WFIRS-P were 0.62 to 0.88 and 0.70 to 0.92 for the WFIRS-S.

The *Behaviour Rating Inventory of Executive Function (BRIEF)* [45] is an assessment of executive function behaviours at home and school for children and adolescents aged 5 to 18. It includes an 86-item parent and teacher report (BRIEF-P, BRIEF-T) and an 80-item self-report (BRIEF-SR). The scales range from 0 = not true to 2 = very true and converted T-scores above 65 indicate executive dysfunction. The inventories contain both a metacognitive (MI) and a behaviour regulation index score (BRI), in addition to a global executive composite score (GEC). We used the GEC index T-score in this study. The inventories have shown good psychometric properties in American and Norwegian children and adolescent populations [46–48].

The *Screen for Child Anxiety-Related Emotional Disorders* (SCARED) [49] is a 41-item self-report screening questionnaire measuring anxiety symptoms in youth. The item scale ranges from 0 = not at all to 2 = often, and a total score ≥ 25 may indicate the presence of an anxiety disorder. The instrument is sensitive to detecting specific and/or comorbid anxiety diagnoses in youth [50]. The Norwegian version has shown excellent internal consistency and convergent validity with other measures of anxiety in a non-clinical population [51]. The Cronbach’s alpha was 0.95 in the current study.

The *Mood and Feelings Questionnaire-short version* (SMFQ) [52] is a 13-item inventory tool that measures depressive symptoms in children and adolescents from 8 to 18 years. The scale ranges from 0 = not true to 2 = true. We used the total score, with higher scores representing more depressive symptoms. In a Swedish clinical population, the SMFQ’s ability to discriminate depression was fair for boys and good for girls. A Norwegian study found the measure to be a fast, practical, and feasible measure to detect depression in school adolescents [53, 54]. The Cronbach’s alpha was 0.93 in the current study.

The *General Perceived Self-Efficacy Scale* [55] is a ten-item one-dimensional scale that is designed to assess belief in one’s ability to cope with a broad range of stressful and challenging demands in life. The items range from 1 = all wrong to 4 = completely right, and a high score represented positive self-efficacy. Studies have found self-efficacy to be a universal construct with high internal consistency across 25 nations, and convergent validity with other similar constructs has been moderate to low [56, 57]. In this study, the Cronbach’s alpha was 0.88.

The *Rosenberg Self-Esteem Scale (RSES)* [58] is a ten-item self-report instrument for evaluating one’s overall sense of worthiness as a person in adolescents and adults. Responses were coded on a 4-point scale ranging from 1 = strongly disagree to 4 = strongly agree. Items 2, 5, 6, 8 and 9 were reversed to yield opposite values, and a high total score indicates positive self-esteem. The scale has exhibited high internal consistency, acceptable criterion validity and discriminant validity, as well as sensitivity to change [59]. In this study, the Cronbach’s alpha was 0.93.

The *Adolescent Sleep-Wake Scale (ASWS)* [60] is a 28-item scale widely used to measure sleep quality in 12 to 18-year-old adolescents. The scale ranges from 1 = always to 6 = never. Eight of the items were reversed for opposite scores. A higher score equals a better quality of sleep. We calculated the mean score in this study. The scale is considered a reliable and valid measure of overall sleep behaviour in a young adult population, with good psychometric properties [60, 61]. The Cronbach’s alpha was 0.70 in the current study.

### Randomisation

A research assistant randomised the participants in a 1:1 ratio (simple randomisation) into one of the treatment arms after the baseline assessments. This was done by a randomization program supplied by the Unit for Applied Clinical Research, a centre of expertise in the Central Norway Health Region. Codes were used to ensure participant confidentiality and anonymity. The participants were not blinded to the treatment condition.

### Statistical analyses and sample size

Previous CBT programs have shown a 5- to 10-point reduction in ADHD-RS IV scale scores post-treatment [16, 25]. Sample size was calculated for a six-point difference, assuming a standard deviation of nine on the ADHD-RS IV, as recommended by Coghill and Seth [62]. With a significance level of 5%, we needed 37 participants in each group to obtain 80% power. To allow for dropouts, we aimed to include 48 participants in each group, for a total of 96. We used mixed models, with the outcome variable as the dependent variable, time point and the interaction between treatment group and time point as fixed effects, and the patient as a random effect. In this way, by not including any systematic main effect on the part of treatment group at baseline, we handled the baseline values of the outcome variable as recommended by Twisk et al. [63]. We did not adjust for any background variables in the main analyses, because we did not have a priori evidence that there are strong prognostic factors that we ought to adjust for. Analyses were based on intention-to-treat (ITT). Separate analyses were conducted for each outcome. Missing data were handled using single imputation on scales using the mean item score if 70% or more of the questions were answered. Otherwise, the outcome of that specific questionnaire for that participant was treated as missing. The normality of residuals was checked via a visual inspection of QQ plots. There were a few residuals for which we were in doubt regarding whether they should be considered outliers. We repeated the three analyses without the four, one and two observations related to these residuals. The results of the analyses were substantially the same (data not shown). Finally, post-hoc subgroup analyses were conducted to explore whether age, IQ, socioeconomic status (SES), the severity of anxiety symptoms or the severity of ADHD symptoms (ADHD-RS IV) would act as a moderator, using the parent-rated ADHD-RS IV total score. This was done by adding the potential moderator and the relevant interactions into the linear mixed models. Statistical analyses were conducted using IBM SPSS Version 25. We report 95% confidence intervals (CIs) where relevant and regard two-sided *p*-values ≤0.05 as significant.

## Results

### Participant attrition and adherence

See Fig. 1. for a flow diagram of the participants in the RCT. Of the 100 participants randomised into the study, 94 (94.0%) completed the post-treatment assessment. The reasons for dropping out of the control group were dissatisfaction with the control condition (*N* = 2) and low motivation (*N* = 1). The reasons for dropping out of the CBT group were lack of motivation to continue with the therapy (*N* = 2). One participant completed the CBT treatment but contracted an illness during the study period, making a post-treatment assessment of ADHD symptom severity impossible (*N* = 1).

Regarding CBT group attendance, 20 participants (43%) attended all twelve sessions, and 39 participants (83%) attended ten or more sessions. Mean attendance was 10.7 sessions (SD 1.4).

### Medication adherence

A majority of the study participants reported good medical adherence (medication ≥ five days a week), at 80.0% in the CBT group and 86.0% in the control group, respectively. Two participants in the CBT group and three in the control group stopped taking their ADHD medication during the trial. Four participants in the CBT group and three in the control group changed their type of ADHD medication during the same period.

### Primary outcomes

Between- and within-group differences are presented in Table 4. No differences were observed between the groups regarding post treatment changes in parent-rated (estimated difference − 0.08, 95% CI, − 2.5 to 2.32, *p* = 0.95), self-rated (estimated difference 1.44, 95% CI, − 1.65 to 4.52, *p* = 0.36) or teacher-rated (estimated difference − 1.51, 95% CI, − 5.1 to 2.0, *p* = 0.40) ADHD symptoms. All three informants reported reduced ADHD symptoms post treatment, with parents and teachers reporting larger symptom reductions than the adolescents. Additional interpretations of the CIs were made to distinguish between negative or inconclusive treatment effects, as recommended by Gewandter et al. [64]. None of the CIs for the ADHD RS-IV parent-, teacher and self-report total scores crossed the 6-point symptom reduction limit, considered a clinically meaningful difference, defined as a 30% symptom reduction from the baseline scores [65]. This strengthens the conclusion of no treatment effect.

**Table 4:** Primary and secondary outcome measures. Descriptive statistics at baseline and post-test, as well as estimated treatment effect (coefficient for the interaction term) from the mixed-model analyses

	CBT Group (*n* = 50)			Control Group (*n* = 50)				Difference (Group x Time)		
Measures	n	Mean	SD	n	Mean	SD	Estimate	95% CI	*P* Value	*Standardized* *effect size*
ADHD RS-IV Parent total score										
Baseline	48	24.19	9.59	49	25.71	8.09				
Post-treatment	45	19.22	8.67	46	20.74	8.52	−0.08^a^	−2.49 to 2.32	.948	−0.009
ADHD RS-IV Inattention score										
Baseline	49	15.12	5.13	49	15.96	5.07				
Post-treatment	46	12.46	4.98	46	13.22	5.45	0.04^a^	−1.50 to 1.57	.963	0.008
ADHD RS-IV Hyperactive score										
Baseline	49	8.98	5.70	50	9.90	5.35				
Post-treatment	46	6.85	5.12	47	7.62	4.79	−0.15^a^	− 1.47 to 1.16	.821	−0.027
ADHD RS-IV Self total score										
Baseline	44	21.55	9.75	47	21.49	10.15				
Post-treatment	44	19.80	9.88	45	18.67	10.21	1.44^a^	−1.65 to 4.52	.359	0.145
ADHD RS-IV Inattention score										
Baseline	47	12.32	4.99	49	11.31	6.28				
Post-treatment	47	11.09	5.50	46	10.13	6.08	0.61^a^	−1.19 to 2.41	.502	0.108
ADHD RS-IV Hyperactive score										
Baseline	46	9.35	6.10	47	9.96	5.14				
Post-treatment	44	8.82	6.17	46	8.67	5.07	0.51^a^	−1.19 to 2.21	.551	0.091
ADHD RS- IV Teacher total score										
Baseline	28	19.07	10.30	36	17.22	8.54				
Post-treatment	28	14.39	9.88	32	12.66	7.23	−1.51^a^	−5.06 to 2.04	.400	−0.160
ADHD RS- IV Inattention score										
Baseline	28	14.29	6.32	37	12.11	6.14				
Post-treatment	30	10.33	7.01	32	9.28	5.15	−1.76^a^	−3.96 to 0.43	.113	**−**0.283
ADHD RS- IV Hyperactive score										
Baseline	34	4.68	5.87	37	5,16	5,03				
Post-treatment	29	3.97	4.29	35	3,51	4,28	−0.31^a^	−2.09 to 1.47	.730	−0.057
Clinical Global Impression Severity										
Baseline	50	3.96	0.53	50	3.92	0.67				
Post-treatment	47	3.38	0.82	47	3.40	0.99	−0.02^a^	−0.31 to 0.26	.883	−0.033
Children’s Global Assess. Scale										
Baseline	50	62.18	6.98	50	62.12	6.82				
Post-treatment	47	61.30	8.66	47	61.04	10.44	0.03^b^	−3.01 to 3.06	.985	0.004
WFIRS-Parent total mean score										
Baseline	44	0.78	0.39	44	0.80	0.38				
Post-treatment	45	0.69	0.39	46	0.73	0.41	−0.01^a^	−0.13 to 0.10	.817	−0.026
WFIRS-Self total mean score										
Baseline	44	0.83	0.49	44	0.82	0.48				
Post-treatment	43	0.70	0.44	45	0.73	0.52	−0.03^a^	−0.15 to 0.09	.599	−0.062
BRIEF-Parent GEC (T-score)										
Baseline	50	66.40	11.18	50	69.64	9.46				
Post-treatment	46	62.67	11.59	47	65.34	10.53	−0.27^a^	−2.30 to 2.46	.844	−0.026
BRIEF-Self GEC (T-score)										
Baseline	50	63.78	11.44	50	64.02	14.78				
Post-treatment	47	61.40	13.17	46	62.24	13.92	−0.02^a^	−3.35 to 3.32	.993	−0.002
BRIEF-Teacher GEC (T-score)										
Baseline	31	77.71	15.87	37	75.05	15.57				
Post-treatment	31	70.97	17.62	33	70.15	15.32	−3.21^a^	−8.10 to 1.68	.195	−0.204
SCARED										
Baseline	45	21.64	14.33	47	22.09	16.45				
Post-treatment	42	18.79	13.52	43	20.01	15.04	.97^a^	−2.92 to 4.85	.622	0.063
Short Mood and Feeling Q.										
Baseline	50	7.96	6.82	49	9.15	6.95				
Post-treatment	47	7.63	6.11	47	7.45	6.42	1.07^a^	−0.89 to 3.03	.284	0.155
General Perceived Self-Effic. Scale										
Baseline	49	27.56	5.22	49	28.04	5.05				
Post-treatment	47	29.21	4.13	47	29.12	5.84	0.46^b^	−1.13 to 2.04	.571	0.090
Rosenberg Self-Esteem Scale										
Baseline	50	28.14	6.57	49	28.64	6.87				
Post-treatment	47	29.47	5.89	47	29.15	6.80	0.70^b^	−0.74 to 2.13	.338	*0.104*
Adolescents’ Sleep-Wake Scale										
Baseline	49	2.76	0.39	49	2.76	0.53				
Post-treatment	46	2.75	0.37	47	2.85	0.49	−0.06^b^	−0.21 to 0.09	.435	*−0.130*

*Note: Baseline* study inclusion *ADHD-RS* Attention-Deficit/Hyperactivity Disorder Rating Scale*, WFIRS* Weiss Functional Impairment Rating Scale, *BRIEF* Behaviour Rating Inventory of Executive Function, *SCARED* Screen for Child Anxiety Related Emotional Disorders, *GEC* General Executive Composite. ^*a*^ a negative difference estimate is in favour of the CBT group and a positive estimate is in favour of the control group*.* ^*b*^a positive difference estimate is in favour of the CBT group and a negative estimate is in favour of the control group. The standardized effect size equals the estimate divided by the average standard deviation at baseline

Supplementary analyses excluding posttreatment data on five participants in the control group receiving extra intervention, gave substantially the same results for all the outcomes (data not shown). Post-hoc subgroup analyses exploring the potential moderating effects of age, IQ, SES, the severity of anxiety symptoms and ADHD symptoms revealed no significant effect on treatment outcome using parent-rated ADHD symptom scores.

### Secondary outcomes

No differences were observed between the CBT group and the control group regarding symptom impairment, functional impairment, executive functions, emotional symptoms, self-efficacy, or self-esteem (see Table 4). Within-group differences revealed reduced symptom severity in both groups on the IE-rated CGI-S symptom severity scale, the parent- and self-rated WFIRS-scales and the BRIEF parent, self- and teacher reports. The IE-rated C-GAS score did not improve post treatment in either group.

## Discussion

Treatment guidelines for ADHD recommend multimodal interventions, including psychoeducation and pharmacological treatment in young people with moderate to severe symptoms [12, 13]. Many patients find this combined treatment insufficient in alleviating their symptoms however, indicating a need for complementary treatments. The NICE guidelines consider CBT as a treatment option for young people with continued impairment after medication [12]. Although, behavioural interventions are considered well established for children with ADHD, this categorization has been limited to organization training for young adolescents (15 years and younger) with ADHD. CBT based interventions directed at adolescents with parent involvement were classified as probably efficacious in a review by Evans et al. [14]. Trials of CBT interventions targeting older adolescents (15 years and older) are limited, however preliminary results from a study of individualised CBT by Sprich et al. [16] and a group CBT by Vidal et al. [25] showed preliminary positive results. Our study thus aimed to assess the efficacy of a group CBT as a follow-up treatment for adolescents who still presented ADHD symptoms and functional impairments, after previously having received a psychoeducational intervention and medication.

In contrast to our hypothesis, the additional group CBT program could not demonstrate an incremental treatment effect as compared to the control condition. Indeed, previous studies of CBT with adolescents found larger post-treatment reductions in ADHD symptoms and improved functional impairment as compared with medicated waitlist controls [16, 25]. In our study, the participants received psychoeducation and pharmacological treatment, interventions recommended by ADHD treatment guidelines, prior to additional CBT. The ADHD symptoms at baseline were thus somewhat lower than in comparable studies [16, 25]. Another reason for the lower baseline scores could be an actual difference in populations (more females, less hyperactivity symptoms and few participants with comorbid ODD). Although our CAP standard intervention may in part explain a lack of treatment effect regarding ADHD symptoms compared to studies including participants with more severe symptoms, it cannot explain the nonsignificant effect of group CBT as compared to the control condition. However, the result is consistent with findings from other studies of youth directed psychosocial treatments without parent involvement [66, 67]. These findings suggest that a more focused and/or individually targeted intervention with the inclusion of parents, similar to the evidence-based treatment programs by Sprich et al. [16], and Sibley et al. [17], could be more effective for this patient group.

However, several questions remain. First, the mean age of our study population was 15.8 years (SD = 1.3), and somewhat younger than the mean age of 17.2 (SD = 1.8) years in a comparable CBT group study by Vidal et al. [25]. Perhaps, the group format is more appropriate for older adolescents or young adults, who are more mature and thus more able to incorporate CBT principles and strategic tools into their daily lives. Second, our treatment program contains eleven modules, with new concepts and skills being introduced over a brief period. Although we consider all the modules relevant when treating adolescents with ADHD and comorbid conditions, such a comprehensive program leaves little time to practice new skills. Based on the adolescents` own report, only one third of the homework assignments was completed. This lower-than-expected completion rate may be explained by too little time to practice new strategies in and between sessions. Because practising new skills is considered a prerequisite for change, this may have contributed to lack of treatment effect. Another hypothesis explaining the low homework adherence may be a more general “lack of motivation” to work between sessions. This pattern was observed across themes and participants and was reported by both group-leaders in checklists and by descriptions from the research assistants talking to the participants between session. These observations suggests a particular challenge working with adolescents with ADHD who often struggle with poor decision making and poor insight into own functioning [68]. The inclusion of more engagement-focused components such as a more systematic exploration of goals and values, a stronger emphasis on motivational interviewing techniques [69] to target out-of-session skills application, and an even stronger emphasis on rewards to improve homework adherence using contingencies based treatment (with the help of parents), could have enhanced treatment engagement [70, 71]. Following this, parent involvement is considered an important treatment component in other programs focusing on helping adolescents with ADHD who struggle with organization, time management and planning for homework assignments [16, 24, 72]. As such, the inclusion of parents in the CBT program could have improved treatment outcome. Third, the parents`- and teachers` BRIEF scores indicated executive dysfunction (T > 65) across groups at baseline. The adolescents reported symptoms just below this cut-off. Although these scores were reduced post-treatment, they still indicated ongoing impairment in both groups. This result pinpoints a need for more extensive training in planning, organisation, and time management over several sessions, as suggested in both school-based behaviour studies [19, 21] and clinic-based CBT studies [17, 24]. Fourth, a structured group format makes it harder to follow up on each participant and individual treatment goals. This could be addressed with the inclusion of an early parallel individual session, working on a case formulation in agreement with the adolescent and thereafter gradually openly sharing each one’s problems in the group. Fifth, our CBT program did not alleviate symptoms of anxiety. This was similar in the study by Vidal and colleagues [25] and suggests a need for more extensive treatment to reduce these symptoms. An RCT by Emilsson et al. [73] found an integrated group and individual CBT program to alleviate both ADHD symptoms and comorbid conditions in adults with ADHD. Such a combined model should be studied in future studies of adolescents with comorbid emotional and behavioural conditions.

This study is to the best of our knowledge, the first RCT to explore the efficacy of a group CBT as follow-up treatment for adolescents with impairing ADHD symptoms who previously received medication and psychoeducational interventions. Strengths of this study include the use of a control condition, the use of blinded evaluators and treatment fidelity ratings. In addition, the use of multiple informants, with self-, parent- and teacher ratings, is considered a strength in terms of the evaluation of the treatment effect. Furthermore, the inclusion of diverse outcome measures covering functional impairment, executive functions, and psychiatric symptoms paints a broader picture of areas in which CBT may have potential treatment effects. Finally, the study was delivered in a real-world setting using clinical staff and covering a total catchment area.

This study has several limitations. First, the large number and low dosage of treatment components caused restricted time to practice each module, which may have affected the treatment outcome. Second, 91% of the population was on pharmacological treatment for ADHD, limiting the study’s generalisability to non-medicated patients. Third, although most study participants fulfilled the diagnostic criteria for an ADHD diagnosis at inclusion, 34% of the participants presented subthreshold ADHD symptoms prior to study inclusion, thus limiting the room for further improvement. Fourth, although the treatment fidelity and deliverance of the CBT program was acceptable, the CBT experience and background of the group leaders varied, which may have affected treatment outcome. Fifth, the socioeconomic status (SES) of the participants was higher than in a typical ADHD population [74, 75], which limits the generalisability of the results to populations with lower SES. Sixth, even though more boys than girls receive an ADHD diagnosis at the CAP clinics [76, 77], girls were easier to recruit for this study, and represented 57% of the population. Boys with comorbid behaviour disorders were particularly hard to recruit, which may suggest that individual- or family-based interventions are more appropriate for this patient group [14]. Seventh, data on homework completion was incomplete, leaving little room for analyses on its impact on treatment effect. Also, there were substantially fewer teacher ratings than self- and parent ratings. This is considered a limitation since their ratings may represent unbiased observations that add to the more proximate and potentially biased parent observations.

Even though our CBT program revealed no overall incremental treatment effect as compared to the control condition, the participants receiving group CBT were positive about joining the program, and dropout rates were low [38]. Future research should examine whether CBT-based treatment programs with parent involvement, focusing on core ADHD symptoms and executive functions that include individualised skills training, contingency management and MI components would be even more effective for this patient group, similar to the clinic-based programs by Sprich [16], Sibley [17] and Boyer [24]. These components are included in an evidence-based psychosocial treatment model for younger adolescent with ADHD [14, 17], but were not included in the current treatment model.

Although treatment guidelines recommend CBT as additional treatment for ADHD in adolescents who still experience functionally impairing symptoms after receiving psychoeducation and medical treatment [12], more research is needed to support the effect of CBT as an adjunct to medication and a historic previous psychoeducation (mean years = 1.8). The guidelines might be clarified to highlight that not all CBT is created equal and that behavioral and cognitive behavioral interventions that include individualized skills training coupled with parent involvement rather than a group program without parent involvement, is the primary evidence-based model for this adolescent age group [16, 17, 21].

## Conclusions

To our knowledge, this RCT is the first study to assess the efficacy of a group CBT as addition to pharmacological treatment and psychoeducational intervention in adolescents with ADHD symptoms and related impairments. In this study the group CBT did not show an incremental effect as a follow-up treatment after a CAP standard clinical intervention. Further studies are warranted to explore the efficacy or effectiveness of a more focused group CBT intervention as addition to medication and psychosocial interventions, and preferably with parents involved. It is also relevant to explore whether more individualised CBT treatment, in a group or individual setting, may be more effective than a standardised program intended to fit all.

## Supplementary Information

**Additional file 1.**

**Additional file 2.**

**Additional file 3.**

## Data Availability

The datasets used in the current study are not publicly available due to privacy policy, but they will be made available from the corresponding author on reasonable request.

## Abbreviations

ADHD: Attention-Deficit/Hyperactivity-Disorder
ADHDRS- IV: ADHD Rating Scale IV
BRIEF-P/−S: The Behaviour Rating Inventory of Executive Function parent report and self-report
CBT: Cognitive Behavioural Therapy
CAP: Child and Adolescent Psychiatry
CAS-CBT: The Competence and Adherence Scale for Cognitive Behavioural Therapy
CGAS: Children’s Global Assessment Scale
CGI-S: Clinical Global Impression Scale for Severity
CI: Confidence Intervals
CONSORT: Consolidated Standards of Reporting Trials
DSM-5: the Diagnostic and Statistical Manual of Mental Disorder 5th edition
ICC: Intraclass Correlation Coefficient
ICD-10: The International Statistical Classification of Disease and Related Health Problems 10th version
IE: Independent evaluator
ITT: Intention-to-treat
Kiddie-SADS-PL: the Schedule for Affective Disorders and Schizophrenia for school age children-Present and Lifetime Version
MI: Motivational interviewing
NICE: National Institute for Health and Care Excellence
RCT: Randomised Controlled Trial
WAIS-IV: the Wechsler Adult Intelligence Scale IV
WISC-IV: the Wechsler Intelligence Scales for Children IV
YBP: Young Bramham programme
