# Supplementary material for: Cognitive behavioural group therapy as addition to psychoeducation and pharmacological treatment for adolescents with ADHD symptoms and related impairments: a randomised controlled trial
Source: BMC Psychiatry. 2022 Jun 2;22:375. doi: 10.1186/s12888-022-04019-6 (PMC9164353; doi:10.1186/s12888-022-04019-6)
Supplement: Supplementary file 1 — Additional file 1. [file 12888_2022_4019_MOESM1_ESM.docx]

**Additional file 1**

Description of the CAP Clinic intervention program before randomization to CBT group treatment or the control condition.

ADHD diagnoses at CAP clinic

**1 hour**: Collaborative meeting at the CAP clinic with a teacher from the patient`s school. A clinician from the CAP clinic, parents of the patient and the primary teacher meet to exchange information from the assessment, receive information about ADHD and discuss supportive measures in the school environment and homework. situation.

**1-2 hours**: Pharmacological treatment. The ADHD patient and parents receive a medical consultation with a child and adolescent psychiatrist with information about pharmacotherapy including possible treatment and side effects. The patient, parents and the psychiatrist evaluate the effect of the medication after a four-week trial period. If the patient experiences no effect or intolerable side effects, one or two other pharmacological options may be explored in order to achieve optimal treatment effect.

**1-2 hours**: Psychoeducation: Information about the ADHD diagnoses to patient and parents from the patient`s clinician (psychiatrist, psychologist or educational specialist); including typical symptoms, causes, advice on family communication, daily structure, diet and a presentation of relevant pharmacological treatment options.

**5 hours**: One-day psychoeducation including lectures on ADHD for parents and teachers:

**2 hours**: Information about symptoms and causes of ADHD and how the symptoms and associated problems may change across different development stages. Information about different treatment options available at the CAP clinic for children and adolescents with ADHD and comorbid conditions. Information about pharmacological treatment by a child and adolescent psychiatrist. Different supportive measures are introduced, such as help with planning and organising, supportive communication and the use of helping aids (digital calendars, alarms on mobile phones).

**1 hour:** Psychosocial and educational interventions in the home environment, and elementary school by a clinical educational specialist. Focus on building a positive relationship between the teacher and pupil, regular daily routines, the use of a daily plan and week plans in school, positioning in the classroom, clear communication/ short messages, regular breaks, help with transitions, the use of visual aids, rewards, and reinforcement, learning by doing, digital learning tools, and considering the need for special education.

**1 hour:** Presentation by a parent of a child with ADHD about personal experiences.

**1 hour:** Presentation by the local ADHD user-organization about rights that comes with the ADHD diagnosis, and social activities available in the local community.

At least one month on stable pharmacological treatment for ADHD

Randomization

Control group (12 weeks) Receiving only one medical control

Group CBT (12 weeks) Including one medical control
