# Supplementary material for: Cognitive behavioural group therapy as addition to psychoeducation and pharmacological treatment for adolescents with ADHD symptoms and related impairments: a randomised controlled trial
Source: BMC Psychiatry. 2022 Jun 2;22:375. doi: 10.1186/s12888-022-04019-6 (PMC9164353; doi:10.1186/s12888-022-04019-6)
Supplement: Supplementary file 2 — Additional file 2. [file 12888_2022_4019_MOESM2_ESM.docx]

**Additional file 2**

Mean daily doses of medication among participants

| ADHD medication and  treatment group | Daily dose (mg)  Mean (SD) | | Daily dose (mg)  Range |
| --- | --- | --- | --- |
| Methylphenidate |  |  |  |
| CBT group n= 29 | 43.0 | 10.3 | 20-65 |
| Control group n= 30 | 48.7 | 10.0 | 27-63 |
| Lisdexamfetamine |  |  |  |
| CBT group n= 8 | 53.8 | 18.5 | 30-80 |
| Control group n=11 | 42.7 | 16.2 | 30-70 |
| Atomoxetine |  |  |  |
| CBT group n=6 | 66.7 | 15.1 | 40-80 |
| Control group n=2 | 57.5 | 10.6 | 50-65 |
| Guanfacine |  |  |  |
| CBT group n= 1 | 1.0 | -- | 1.0 |
| Control group n=3 | 3.7 | 1.5 | 2-5 |
| **Note**: ADHD = attention-deficit hyperactivity disorder | | | |
