# Supplementary material for: Cognitive behavioural group therapy as addition to psychoeducation and pharmacological treatment for adolescents with ADHD symptoms and related impairments: a randomised controlled trial
Source: BMC Psychiatry. 2022 Jun 2;22:375. doi: 10.1186/s12888-022-04019-6 (PMC9164353; doi:10.1186/s12888-022-04019-6)
Supplement: Supplementary file 3 — Additional file 3. [file 12888_2022_4019_MOESM3_ESM.docx]

**Additional file 3**

**Background of the CBT group program**

The CBT program was an adaptation of the CBT program of Young and Bramham (2012), which was developed to treat adolescents and adults with ADHD by providing cognitive behavioural therapy (CBT) to treat core symptoms of the condition and its associated problems. The program consists of stand-alone modules that can be delivered in individual, or a group format. We developed the CBT program in collaboration with Dr Susan Young to fit an adolescent population in a group format (90-minute sessions for 12 weeks).

The CBT program has two primary aims; to encourage people with ADHD to change their environment and optimize functioning (to change from the outside in), and to develop psychological strategies for adaptive functioning (to change from the inside out).

The program includes psychoeducation to inform the client about the diagnosis, treatment and prognosis of the disorder and therapeutic techniques (cognitive behavioural therapy, cognitive remediation, restructuring and reframing, rationalization) to address core symptoms, comorbid and associated problems.

Each module (or session in this RCT) follows a standard format providing a general introduction to the topic, followed by a group discussion, where the adolescents present their experienced difficulties or functional impairment related to this theme. The group leaders use handouts to assess the individual problems of the participants (self-ratings), and different treatment strategies including specific cognitive behavioural strategies and techniques that address the problems are presented for the group. Next, strategies are rehearsed either individually or in pairs, and then shared and discussed with the group. At the end of each session, all participants get home assignments related to the session`s topic. They write down their targeted assignment to rehearse and practice new skills until the next session. The next session starts with a follow-up of the weeks` assignments, where each participant presents their experience practicing the new skills with the group. Potential obstacles to practice or achieve the new goal are addressed.

Coaching: Each week a research assistant telephoned the participants to check if they had done their week assignment or experienced any difficulties with the assignment. The “coach`s” role was to motivate the participant and address ambivalence.

**The content of the CBT group program**

Background and treatment

**Session 1:**  Orienting participants to the program, including content, structure, and the basic CBT principles. Participants receive psychoeducation about ADHD and write down individual treatment goals.

Core symptom modules

**Session 2:** Attention: Various forms of attention and the impact of motivation, anxiety and stress are introduced and discussed. Various attention control strategies are presented and rehearsed in session. We introduce external strategies that may be applied to adapt to the environment to minimize distraction and introduce strategies to optimize performance such as goalsetting, breaks, incentives, and rewards.

**Session 3:**  Memory: The various memory systems are introduced, and individual memory problems are identified (handouts). External memory strategies (such as the use of diaries, electronic devices and alarms) and internal memory strategies (such as repetition, rehearsal, visual cue and use of mnemonics) are presented. Memory games and exercises are practiced within the group.

**Session 4:**  Organizing and time-management: Consequences of dysfunctional planning and time-management are discussed. A methodical approach to make plans is presented by reviewing goals for a set time period (short and long-term), listing activities, devising a schedule, prioritizing activities, and planning breaks and rewards. We also include methods for avoiding pitfalls, such as ways of maintaining attention on task, advice regarding reviewing priorities and avoiding procrastination.

**Session 5:**  Impulsivity: We address behaviours that are often closely linked to a low tolerance of boredom, feelings of frustration, a preference for short-term rewards and an inability to delay gratification. Consequences of having low self-control are introduced and discussed. Self-monitoring techniques are introduced to identify situations in which adolescents are vulnerable to responding in an impulsive way. Various impulse control strategies are introduced (including stop-and-think techniques, self-talk and distraction techniques) and rehearsed in roleplays.

Comorbid and associated problem modules

**Session 6:** Problem solving: The participants learn how to define problems, generate solutions, evaluate solutions, and consider alternatives. A methodology for choosing solutions is introduced through the rehearsal of solution to evaluate consequences. We rehearse in session, and finally, we evaluate the level of success.

**Session 7:** Anxiety: We address the generalized and social anxiety problems commonly seen in adolescents with ADHD. The CBT principles from session 1 are repeated (the three-legged table is reintroduced) and we present methods to re-interpret common responses to anxiety by evaluating thoughts, feelings, behaviors, and bodily reactions. A version of the cognitive behaviour model of panic (Clark, 1986) is presented and strategies for intervention, including relaxation and breathing exercises. We suggest ways to overcome avoidance and increase confidence by applying techniques of graded exposure, systematic desensitization, and behavioural experiments.

**Session 8:** Low mood and depression/ sleep management: We introduce a cognitive model of depression (Beck 1976) that incorporates negative thinking and thinking errors common to adolescents with ADHD. We suggest how to break the negative cycle and introduce strategies that include activity scheduling, techniques to challenge negative automatic thoughts and introduce positive self-talk. Psychoeducation about sleep and sleep strategies including advice on sleep hygiene and relaxations techniques are introduced.

**Session 9:** Interpersonal relationships and communication: We address disruption to interpersonal relationships that may be a problem for adolescents with ADHD. We focus and rehearse verbal and nonverbal communication strategies, and how to modify and regulate social behavior in different social settings.

**Session 10:** Frustration and anger management: Consequences of bad anger management are discussed. We introduce various management strategies, including self-talk, distraction techniques, reframing the situation and relaxation.

The future module

**Session 11-12:** Preparing for the future: We present and discuss the challenges of having ADHD in the transition to young adulthood. We summarize some of the techniques introduced in the previous modules, and each participant sum up those strategies they found the most helpful. We discuss the participants’ future goals and which skills and support networks that can be used to achieve them.

Each participant receives positive feedback on their achievements related to individual treatment goals and their contribution in the group by the group members and the group leaders.

**References**

Beck A.T. Cognitive Therapy and the Emotional Disorders, New York, International Universities Press, 1976.

Clark, D.M. A cognitive approach to panic. Behaviour research and Therapy; 24, 461-470, 1986.

Young S, Branham, J. Cognitive-Behavioural Therapy for ADHD in Adolescents and Adults- A Psychological Guide to Practice. 2nd. ed. Wiley-Blackwell; 2012.
